# Co-culture of STRO1 + human gingival mesenchymal stem cells and human umbilical vein endothelial cells in 3D spheroids: enhanced *in vitro* osteogenic and angiogenic capacities

**DOI:** 10.3389/fcell.2024.1378035

**Published:** 2024-05-06

**Authors:** Yushan Liu, Pei Chen, Tengfei Zhou, Jincheng Zeng, Ziyi Liu, Ruijie Wang, Yiwei Xu, Wuwei Yin, Mingdeng Rong

**Affiliations:** ^1^ Department of Periodontology and Implantology, Stomatological Hospital, School of Stomatology, Southern Medical University, Guangzhou, China; ^2^ Guangdong Provincial Key Laboratory of Medical Molecular Diagnostics, Guangdong Key Laboratory of Medical Bioactive Molecular Developmental and Translational Research, Guangdong Medical University, Dongguan, China; ^3^ Guangzhou Regenerative Medicine and Health Guangdong Laboratory, CAS Key Laboratory of Regenerative Biology, Guangzhou Institute of Biomedicine and Health, Chinese Academy of Science, Guangzhou, China

**Keywords:** Gingival mesenchymal stem cells, human umbilical Vein endothelial cells, STRO1, three-dimensional culture, stem cell spheroids, osteogenic differentiation, angiogenic differentiation

## Abstract

Stem cell spheroid is a promising graft substitute for bone tissue engineering. Spheroids obtained by 3D culture of STRO1+ Gingival Mesenchymal Stem Cells (sGMSCs) (sGMSC spheroids, GS) seldom express angiogenic factors, limiting their angiogenic differentiation *in vivo*. This study introduced a novel stem cell spheroid with osteogenic and angiogenic potential through 3D co-culture of sGMSCs and Human Umbilical Vein Endothelial Cells (HUVECs) (sGMSC/HUVEC spheroids, GHS). GHS with varying seeding ratios of sGMSCs to HUVECs (GHR) were developed. Cell fusion within the GHS system was observed via immunofluorescence. Calcein-AM/PI staining and chemiluminescence assay indicated cellular viability within the GHS. Furthermore, osteogenic and angiogenic markers, including ALP, OCN, RUNX2, CD31, and VEGFA, were quantified and compared with the control group comprising solely of sGMSCs (GS). Incorporating HUVECs into GHS extended cell viability and stability, initiated the expression of angiogenic factors CD31 and VEGFA, and upregulated the expression of osteogenic factors ALP, OCN, and RUNX2, especially when GHS with a GHR of 1:1. Taken together, GHS, derived from the 3D co-culture of sGMSCs and HUVECs, enhanced osteogenic and angiogenic capacities *in vitro*, extending the application of cell therapy in bone tissue engineering.

## 1 Introduction

Stem cell-based tissue engineering for bone regeneration is progressively investigated in preclinical trials to address periodontal bone loss caused by trauma, tumors, inflammation, congenital anomalies, and others (Larsson et al., 2016; Fu et al., 2021). Bone regeneration is a slow and intricate physiological process that necessitates a robust blood supply to ensure both the early survival of grafts and the activation of their regenerative potential (Diomede et al., 2020). Conventionally, a single type of stem cell cultured in a 2D environment is chosen as seed cells for bone tissue engineering, dependent on their proliferative and osteogenic differentiation on scaffold materials to facilitate bone defect repair (Hao et al., 2017). 3D culture-derived stem cell spheroids have recently gained traction in tissue repair and regenerative therapies (Kim et al., 2023). Compared with 2D culture induction, stem cell spheroids cultured in 3D can self-assemble into spheroids, better simulating the interactions between cells and the extracellular matrix (ECM) (Ryu et al., 2019), thereby exhibiting superior cellular behaviors such as higher cell viability, phenotypic stability, differentiation potential, and protein secretion function (Yin et al., 2016; Moritani et al., 2018; Yen et al., 2023). Therefore, these potent regenerative stem cell spheroids harbor significant potential when employed as seed cells in bone tissue engineering.

Mesenchymal stem cells (MSCs) are a group of stem cells with high self-renewal capacity and multipotent differentiation potential (Margiana et al., 2022). Various MSC populations are abundantly found in dental tissues such as dental pulp, periodontal ligament, and gingiva (Sharpe, 2016). *In vitro* experiments reveal that these dental MSCs can differentiate into bone, cartilage, and fat cells, exhibiting impressive performance in proliferation, differentiation, and preservation of stemness, outperforming the commonly used stem cells like Bone Marrow Mesenchymal Stem Cells (BMSCs) and Adipose Mesenchymal Stem Cells (ADSCs) in the realm of bone tissue engineering (Huang et al., 2009; Kunimatsu et al., 2018). Gingival Mesenchymal Stem Cells (GMSCs) are derived from the neural crest during early embryonic development, thus preserving a partial capacity for progenitor cell differentiation (Kim et al., 2021). Following specific induction *in vitro*, they have the potential to differentiate into various cell types, including neural cells, chondrogenic cells, and myogenic cells, among others (Wang et al., 2011; Ansari et al., 2016; Zhang et al., 2021). GMSCs are convenient to isolate and obtain and exhibit robust stemness stability following prolonged *in vitro* culture (Jin et al., 2015; Xing et al., 2019). Recent studies suggest that GMSCs secrete certain proteins exhibiting anti-inflammatory, anti-apoptotic, and immune-regulatory properties (Zhang et al., 2012; Yang et al., 2013; Wang et al., 2020). Remarkably, GMSCs demonstrate superior differentiation potential to other mesoderm-derived dental stem cells, such as Periodontal Ligament Stem Cells (PDLSCs) and Dental Pulp Stem Cells (DPSCs) (Tomar et al., 2010; Gao et al., 2014; Angelopoulos et al., 2018). However, primary GMSCs, isolated from gingiva tissue, are not homogeneous and potentially exhibit variances in stemness levels within the cell populations (Diar-Bakirly and El-Bialy, 2021). The expression of the progenitor cell marker STRO1 is a pivotal indicator of stemness, and MSCs with high expression of STRO1 usually exhibit a higher osteogenic differentiation potential (El-Sayed et al., 2015). Therefore, it is significant to enrich and separate a subpopulation of GMSCs with high expression of STRO1 (i.e., sGMSCs) to enhance their regenerative potential and therapeutic effects. Nonetheless, the construction of vascularized bone tissue grafts is integral for early stem cell survival and activation of regenerative potential. Studies, both *in vitro* and *in vivo*, suggest that although GMSCs can partially repair bone defects through osteogenic differentiation, they hardly express hematopoietic markers like CD14, CD31, CD34, and CD45, resulting in inadequate neovascularization following transplantation *in vivo*, and ultimately impinging the efficiency of bone regeneration and remodeling (Jin et al., 2015).

HUVECs, derived from the umbilical cord, are commonly used in angiogenic research. They exhibit high expression of angiogenic factors such as CD31, CD34, and VEGFA, and have been shown to promote the cellular viability and differentiation potential of MSCs(Carvalho et al., 2019; Heo et al., 2019). Current research indicates that co-culturing MSCs and HUVECs in 3D allows long-term *in vitro* proliferation while preserving genetic and phenotypic stability (Piard et al., 2019). Therefore, it can be reasonably inferred that 3D co-cultured GHS could function as ideal “seed cells” in bone tissue engineering. Incorporating HUVECs into the co-culture may help compensate for the deficiency of GS in terms of angiogenic differentiation and potentially form vessel networks in the transplanted region. However, the detailed construction aspects of MSC/HUVEC spheroids for osteogenic differentiation, such as composition ratio, induction time, and underlying mechanisms, are still lacking in current research. Consequently, by exploring these key factors, our research introduced a novel strategy based on 3D GHS for developing bone tissue engineering grafts with osteogenic and angiogenic potential. This expands the applicability of cell therapy in the field of periodontal bone regeneration and lays a foundation for subsequent *in vivo* verification of GHS transplantation.

## 2 Materials and methods

### 2.1 Isolation and culture of human GMSCs

GMSCs collection adhered to the Declaration of Helsinki and associated regulations. Consent forms were signed by all volunteers, with ethical approval granted by the Ethics Committee of the Stomatological Hospital of Southern Medical University (Approval No.: EC-CT-[2022]41).

The isolation and culture of GMSCs followed a modified protocol from Zhang et al. (Zhang et al., 2009). Gingival tissues, acquired from healthy individuals aged 23–30 years, were carefully segmented into 1–2 mm^3^ pieces and thoroughly rinsed with sterile PBS. Subsequently, the tissues were incubated with Dispase II (Solarbio, Beijing, China) overnight at 4 °C to separate the epithelial layer from the underlying connective tissue. Collagenase Type IV (Solarbio) digestion was then carried out at 37 °C for 1 h. The centrifuged pellet was then resuspended in Dulbecco’s Modified Eagle Medium supplemented (DMEM; Gibco, MA, USA) with 10% fetal bovine serum (FBS; Gibco) and 1% penicillin-streptomycin (P/S; Gibco). The cells were cultured in T25 flasks at 37 °C with 5% CO_2_. Passages were performed using 0.25% trypsin when the cell confluence reached 70%–80%. Experiments were conducted using cells at passages 3-6.

### 2.2 Enrichment and culture of sGMSCs

The P3 GMSCs were suspended and quantified in pre-cooled Magnetic Activated Cell Sorting (MACS) buffer, consisting of DPBS with 0.5% BSA and 2 mM EDTA. After centrifugation, the supernatant was discarded. PE-conjugated STRO1 antibody (Abcam, Cambridge, UK) was introduced to the cells and incubated in the dark for 2 h. Following incubation, MACS anti-PE magnetic beads (Miltenyi Biotec, Bergisch Gladbach, Germany) were added. After incubating for 30 min, the cells not bound to the beads were discarded by a washing step, followed by resuspension and transfer into an MS separation column. Magnetic separation was performed to collect the positively sorted GMSCs (sGMSCs). The isolated sGMSCs were utilized for experiments or seeded in 6-well plates for temporary storage and culture.

### 2.3 Characterization of sGMSCs

#### 2.3.1 Flow cytometry

sGMSCs were respectively labeled with rabbit anti-human CD14, CD31, CD34, CD45, CD73, CD90, and CD105 (Abcam) according to the manufacturer’s instructions. A rabbit anti-human IgG antibody (Abcam) served as a negative control. The tube was incubated in the dark at room temperature (RT) for 2 h. After incubation, cells were washed three times with PBS to remove unbound antibodies. Subsequently, the cells were resuspended and analyzed using a Beckman CytoFLEX flow cytometer (Beckman Coulter, IN, USA) with appropriate settings and compensation adjustments. The negative control was utilized to establish the negative boundary, while the fluorescence intensity and number of positive cells were measured.

#### 2.3.2 Osteogenic differentiation

Both sorted sGMSCs and unsorted primary GMSCs (pGMSCs) were seeded at a density of 1×10^5^ cells per well in a 12-well plate containing DMEM. After 72 h, the culture medium was replaced with osteogenic induction medium consisting of DMEM supplemented with 20% FBS, 1% P/S, 10 nM Dexamethasone, 100 mM L-ascorbic acid, and 10 mM β-glycerophosphate (Beyotime, Shanghai, China). The induction medium was replaced every 48 h. Following 21 days of continuous induction, the cells were stained with Alizarin Red S staining solution (Solarbio) at RT for 1 h. Osteogenic differentiation was observed under a microscope (Leica, Mannheim, Germany).

#### 2.3.3 Adipogenic differentiation

After 3 days of seeding, the culture medium was replaced with adipogenic induction medium consisting of DMEM supplemented with 20% FBS, 1% P/S, 1 nM Dexamethasone, 10 μg/mL Insulin, 100 μM Indomethacin, and 0.5 mM IBMX (Beyotime). The induction medium was replaced every 48 h. Following 14 days of continuous induction, the cells were fixed with 4% PFA (Solarbios) for 15 min. Subsequently, the cells were stained with Oil Red O staining solution (Solarbios) at RT. Lipid droplets were observed under a microscope (Leica).

### 2.4 Construction and culture of GHS system in different HUVECs ratios

HUVECs (Cyagen Biosciences Inc., CA, USA) were cultured in Endothelial Growth Medium-2 (EGM-2; Lonza, MA, USA). Before their integration in a 3D co-culture, sGMSCs and HUVECs were collected by trypsin. The osteogenic induction medium in the GS and GHS systems is composed of a combination of EGM-2 and DMEM osteogenic media tailored to match the cell proportions. The induction medium was renewed every 48 h to maintain optimal nutrition for the cellular spheroids.

As a control, sGMSCs and HUVECs were respectively cultured in ultra-low attachment 6-well plates (Corning, NY, USA) at a density of 1×10^6^ cells per well to assemble GS and HUVEC Spheroids (HS). To form GHS with varying ratios, single-cell suspensions of sGMSCs and HUVECs were arranged at a total cellular density of 1×10^6^ cells per well, with GHR of 5:1, 4:1, 3:1, 2:1, and 1:1. These mixed single-cell suspensions were then propagated in ultra-low attachment 6-well plates and subjected to unbroken osteogenic induction for 7–14 days. The morphology of the generated GS and GHS were inspected under a microscope 12–48 h post-seeding, and their diameters were quantified using ImageJ (n = 3).

### 2.5 Analysis of cell fusion and cell viability within the GHS system

#### 2.5.1 Cell fusion in the GHS system

Cell distribution within GHS, GS, and HS were ascertained by immunofluorescence staining. Low-speed centrifugation was employed to separate the supernatant and protect spheroid integrity. Samples were collected, washed thrice with DPBS on an orbital shaker, and fixed with 4% PFA for 2 h. Before immunostaining, tissue clearing reagent CUBIC-L (TCI, Tokyo, Japan) was added to the fixed spheroids to degrease and decolorize them for 12 h. Samples were then blocked with 3% BSA for 2 h, followed by overnight incubation at 4°C with a primary antibody solution containing anti-human CD31 (Abcam) and STRO1 (Santa Cruz, TX, USA) antibodies. Alexa Fluor 488 and 594 labeled secondary antibodies (Abcam) were added the next day and incubated for 1 h in darkness. After washing thrice, the nuclei were counterstained with DAPI (Solarbio). The distribution of STRO1 and CD31 was analyzed using a laser confocal microscope (Leica).

#### 2.5.2 Cell viability in the GHS system

sGMSCs and HUVECs were processed and combined to yield 100 μL single-cell suspension. Subsequently, they were cultured in ultra-low attachment 96-well plates (Corning) at a total density of 1×10^4^ cells per well to form GS and GHS with varied ratios (including GHR of 5:1, 4:1, 3:1, 2:1, and 1:1.). After 7-day osteogenic induction, the culture plates were equilibrated to RT. We then utilized the CellTiter-Lumi Steady Plus Luminescent Cell Viability Assay Kit (Beyotime) to measure the ATP content in the cell spheroids. Afterward, luminescence intensity was measured using a microplate reader (Tecan, CA, USA).

### 2.6 Maintenance time of cell viability within the GHS system

To determine the duration of cell viability within the GHS system and deduce the optimal duration for osteogenic induction, Calcein-AM/PI staining (Beyotime) and CCK-3D assay (Beyotime) were performed on GS and GHS with a GHR of 1:1. Cell viability within the GHS and GS was visualized using Calcein-AM/PI staining and laser confocal microscopy on days 1, 3, 5, 7, 9, 11, and 13. The fluorescence intensity of live and dead cells within the spheroids was quantified using ImageJ (n = 3). CCK-3D assay was used to measure the relative cell viability within GHS and GS. The samples from the GHS on day 1 were marked as the control group. The cell spheroids were incubated with CCK-3D reagent in the dark at 37°C for 2 h, and the absorbance at 450 nm was measured using a microplate reader (Tecan).

### 2.7 Osteogenesis and angiogenesis potential of GHS system with different cell ratios

#### 2.7.1 qRT-PCR assay

To contrast the expression of osteogenic and angiogenic markers in spheroids with varying HUVEC ratios, RNA extraction was performed on GS and GHS samples from day 3, 7, and 14 of osteogenic induction. Total RNA was obtained using an RNeasy Plus Mini Kit (Qiagen, Hilden, Germany), and the resulting RNA was used to synthesize cDNA using the PrimeScript RT Master Mix (Takara, Shiga, Japan). Quantitative real-time polymerase chain reaction (qRT-PCR) was performed using the SYBR Green Premix Pro Taq HS qPCR Kit (AGBio, Hunan, China) and conducted on the LightCycler 96 System (Roche, Basel, Swiss). *β*-actin served as an internal control, and relative gene expression levels were normalized using the 2^−ΔΔCT^ method. Table 1 provides the primer sequences utilized in the study.

**TABLE 1:** Primer sequences used for qRT-PCR.

Genes	Primers sequence
*β*-actin	Forward:5′ CAT​GTA​CGT​TGC​TAT​CCA​GGC
	Reverse: 5′ CTC​CTT​AAT​GTC​ACG​CAC​GAT
Runx2	Forward:5′ CCG​CCT​CAG​TGA​TTT​AGG​GC
	Reverse: 5′ GGG​TCT​GTA​ATC​TGA​CTC​TGT​CC
OCN	Forward:5′ CCT​CAC​ACT​CCT​CGC​CCT​ATT
	Reverse: 5′ GGT​CAG​CCA​ACT​CGT​CAC​AG
ALP	Forward:5′ CCA​GGG​CTG​TAA​GGA​CAT​CG
	Reverse: 5′ GCT​CTT​CCA​GGT​GTC​AAC​GA
CD31	Forward:5′ GAG​TCC​TGC​TGA​CCC​TTC​TG
	Reverse: 5′ ACA​GTT​GAC​CCT​CAC​GAT​CC
VEGFA	Forward:5′ TGC​GGA​TCA​AAC​CTC​ACC​A
	Reverse: 5′ CAG​GGA​TTT​TTC​TTG​TCT​TGC​T

#### 2.7.2 Western blot assay

Cell spheroids were subjected to low-speed centrifugation to separate the culture medium and washed thrice with DPBS. Proteins were extracted using RIPA buffer supplemented with Phosphatase and Protease Inhibitor Cocktails (Beyotime) and quantified utilizing a BCA kit (Solarbio). Proteins were resolved on a 12% SDS polyacrylamide gel and transferred onto a PVDF membrane (Millipore, MO, USA) using a wet transfer process. The membrane was then incubated with primary antibodies against *β*-actin, CD31, VEGFA, RUNX2, ALP, and OCN overnight at 4°C. The following day, the membrane was exposed to an HRP-conjugated secondary antibody, imaged using chemiluminescent reagents (Beyotime).

### 2.8 Statistical analysis

Outliers were excluded, and the data are presented as mean ± standard deviation. Data analysis and visualization were separately conducted via SPSS 23.0 and GraphPad Prism 9.0. Group differences were analyzed using one-way analysis of variance (ANOVA) with Tukey’s multiple comparisons, and Student’s two-tailed unpaired *t-test* was performed when comparing two groups. *p*-value of less than 0.05 (*p < 0.05), 0.01 (**p < 0.01), or 0.001 (***p < 0.001) was considered statistically significant for intergroup differences.

## 3 Results

**FIGURE 1:**
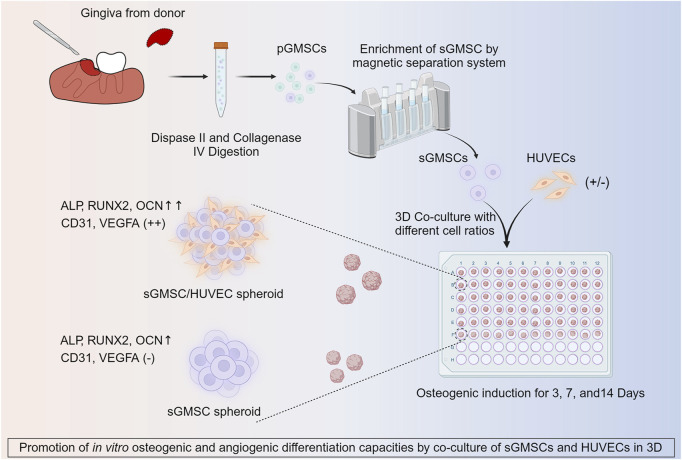
Illustration of co-culture of HUVECs with sGMSCs in 3D for enhancing *in vitro* osteogenic and angiogenic capacities.

The study design is depicted in Figure 1.

### 3.1 Characterization and differentiation ability of sGMSCs

**FIGURE 2:**
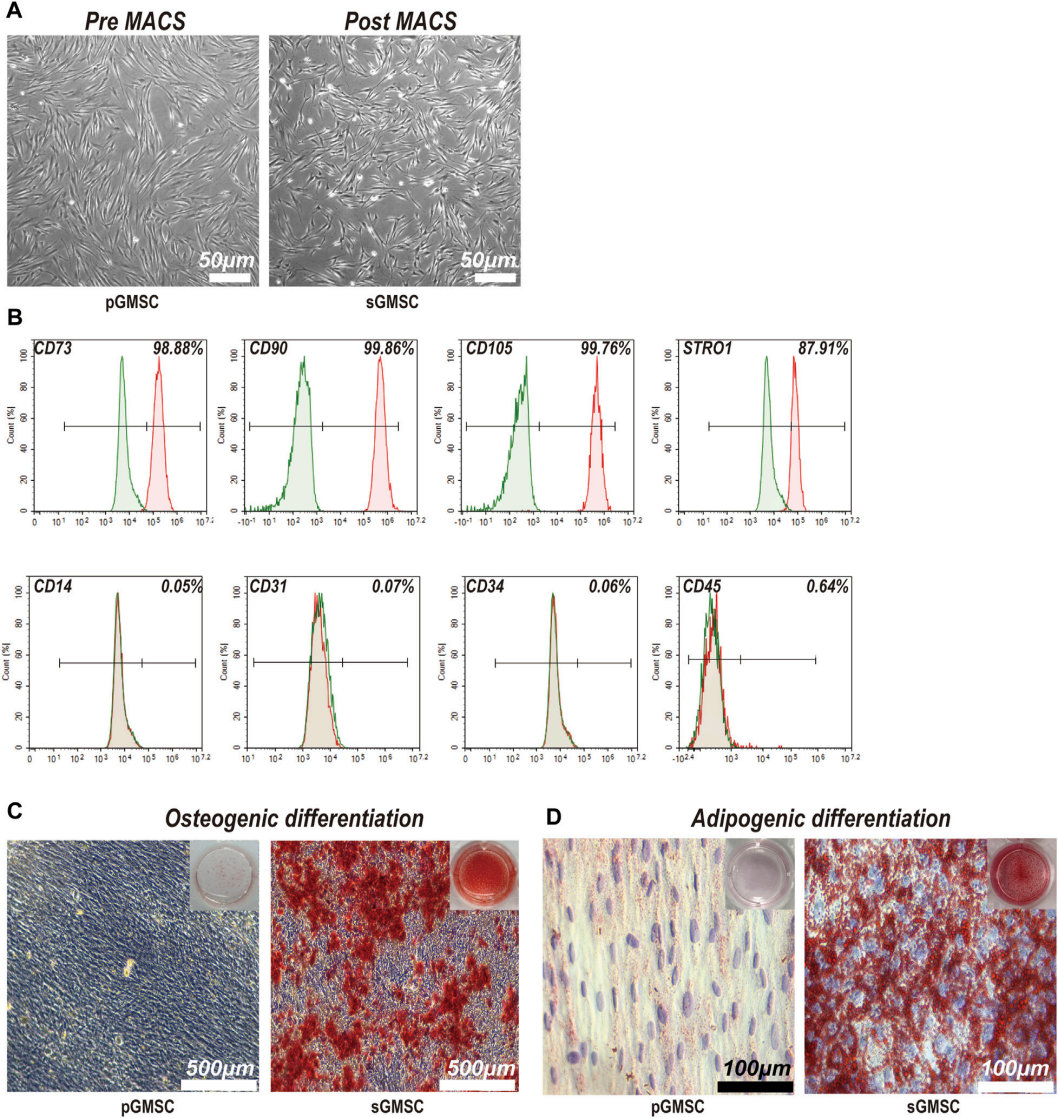
Characterization and differentiation capacities of sGMSCs. **(A)**. Scale bar, 50 μm) pGMSCs and sGMSCs. **(B)** Expression of surface markers on sGMSCs. **(C)**. Scale bar, 500 μm) Alizarin Red S staining of pGMSCs and sGMSCs for osteogenic differentiation. **(D)**. Scale bar, 100 μm) Oil Red O staining of pGMSCs and sGMSCs for adipogenic differentiation.

The pre-MACS separating pGMSCs displayed a spindle-like form in monolayer culture. Post-MACS separation, sGMSCs adopted a polygonal or spindle-like appearance with multiple pseudopodia extending toward the adjacent area (Figure 2A). sGMSCs attain 80%–90% confluency within 3 days.

Immediate flow cytometry analysis of the sorted sGMSCs (Figure 2B) exhibited high expression of mesenchymal stem cell markers CD73 (98.88%), CD90 (99.86%), and CD105 (99.76%), while the progenitor marker STRO1 (87.91%) was observed. The expression of CD14 (0.05%), CD31 (0.07%), CD34 (0.06%), and CD45 (0.64%) remained low. These results affirm sGMSCs as a mesenchymal stem cell population.

After 21-day osteogenic induction, pGMSCs and sGMSCs were stained with Alizarin Red S. The non-sorted pGMSCs showed negligible osteogenic differentiation, whereas the sorted sGMSCs exhibited plentiful calcium deposits (Figure 2C). Moreover, following a 14-day adipogenic induction, Oil Red O staining indicated fewer lipid droplets in non-sorted pGMSCs, whereas sorted sGMSCs manifested a noticeable accumulation (Figure 2D). In conclusion, sGMSCs showed greater osteogenic and adipogenic differentiation potential than pGMSCs, likely attributable to the increased expression of progenitor cell markers, particularly STRO1, post-sorting.

### 3.2 Construction and morphology of GHS

**FIGURE 3:**
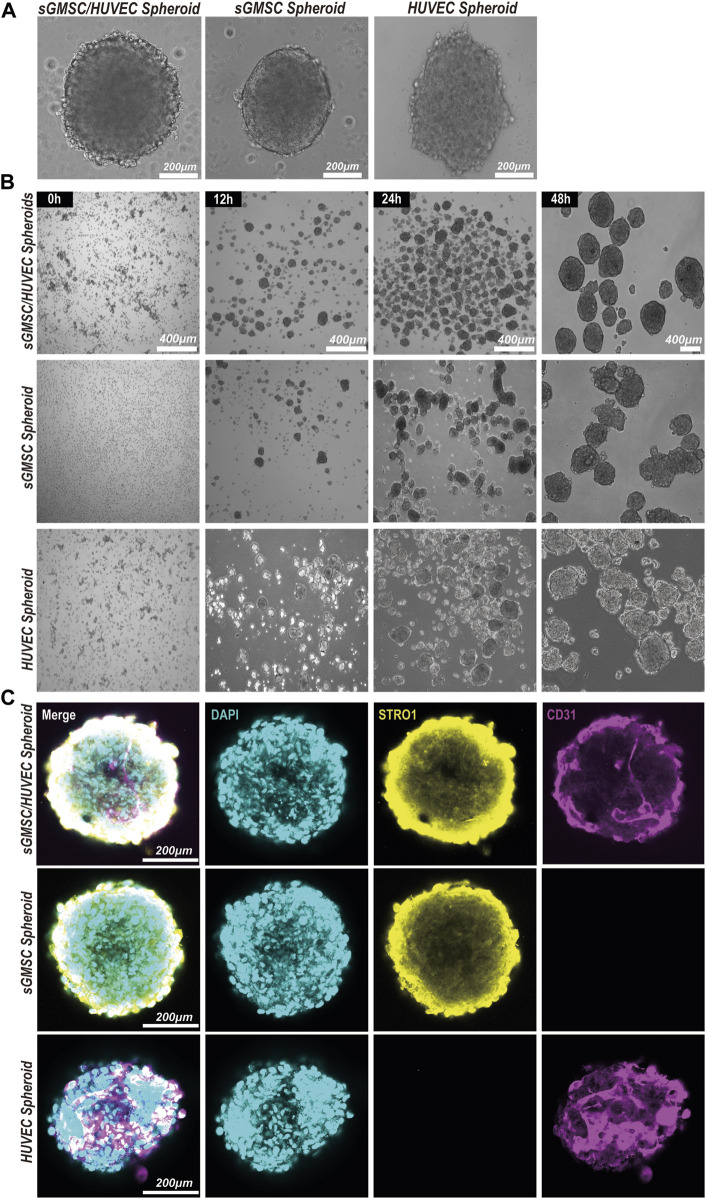
Construction and morphology of GHS. **(A)**. Scale bar, 200 μm) Morphology of GS, HS, and GHS. **(B)**. Scale bar, 400 μm) GHS, GS, and HS formed mature and regular spheroids within 48 h of co-culturing. **(C)**. Scale bar, 200 μm) Immunofluorescence staining of cell-specific markers on day 7 of osteogenic induction. DAPI staining for nuclei (blue), STRO1 staining for sGMSCs (yellow), and CD31 staining for HUVECs (magenta) are shown.

An evaluation of the morphological formation and maturation timeline of GHS was conducted, contrasting these attributes with GS and HS. All three groups formed 3D spheroids within a similar timeframe of 24 h under matrix-free conditions (Figure 3A). However, differences were observed in morphology and diameter: GS presented a smoother edge with a smaller diameter of approximately 374.32 ± 68.91μm; HS showed a spindle shape with more cellular protrusions and a larger diameter of approximately 637.39 ± 130.22μm; GHS formed fairly regular spherical structures with some cellular protrusions, possibly representing unfinished HUVEC contact, measuring approximately 563.63 ± 61.09 μm in diameter. After a 48-h co-culture period, most GHS matured morphologically, forming multiple smooth-edged spheroids (Figure 3B).

The distributions of sGMSCs and HUVECs within 3D spheroids during the seventh day of culture for GHS, GS, and GHS were evaluated by conducting immunofluorescence staining with double labeling of STRO1 and CD31. Observations revealed that in GS, STRO1-positive sGMSCs were evenly distributed with no CD31 fluorescence signal, given that sGMSCs do not express CD31. However, there was a uniform distribution of CD31-positive HUVECs fluorescence signals in HS, while STRO1 fluorescence signals were negative. Conversely, in GHS, the fluorescence signals of high STRO1-expressing sGMSCs and high CD31-expressing HUVECs were uniformly distributed, demonstrating an approximate spherical morphology (Figure 3C). This supported the effectiveness of sGMSCs and HUVECs fusion into cellular spheroids in 3D co-cultures.

### 3.3 HUVECs enhance cell viability within the GHS system

**FIGURE 4:**
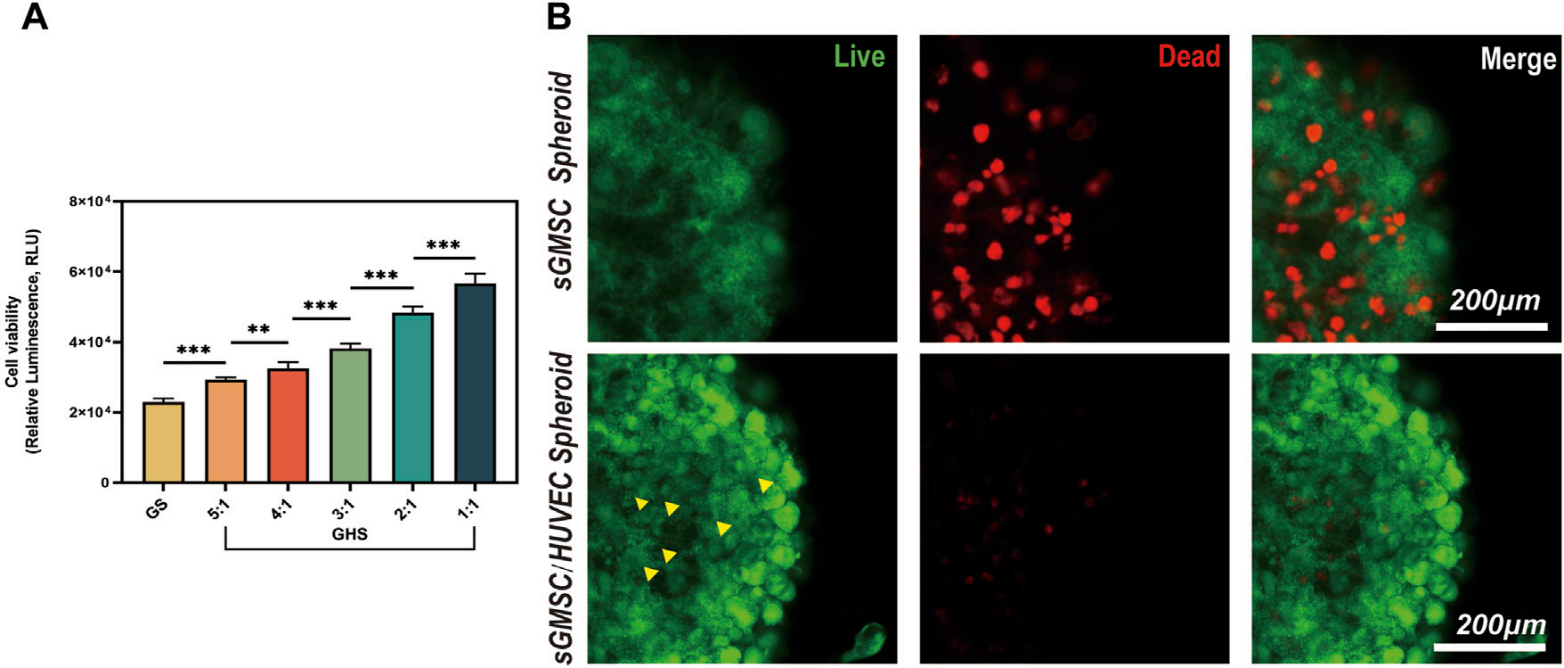
HUVECs promote cell viability and maintain the stability of the GHS system. **(A)** Osteogenic induction of GHS with different cell ratios on day 7. (Student’s two-tailed unpaired *t-test*, *p < 0.05, **p < 0.01, ***p < 0.001). **(B)**. Scale bar, 200 μm) Local magnification of Live/Dead cell staining on day 7 of GS and GHS osteogenic induction (green: live cells; red: dead cells; yellow triangles: dot-like dead cells).

The adenosine triphosphate (ATP) level is directly correlated with cellular viability. After confirming the successful fusion of sGMSCs and HUVECs in the 3D co-culture system, using GS as a control, we varied the ratio of HUVECs in GHS (including GHR of 5:1, 4:1, 3:1, 2:1, and 1:1.) during a 7-day osteogenic induction. ATP levels within GHS with different GHR demonstrated that the incorporation of HUVECs augmented cellular viability within the GHS system. Significant statistical differences were observed among the various groups, and as the ratio of HUVECs increased, the viability of the GHS system also increased. When GHR was 1:1, the cellular viability within the GHS system was significantly higher than that of GS (Figure 4A).

To further corroborate the enhancing impact of HUVECs on cellular viability within the GHS system, both GS and GHS, osteogenic induced for 7 days, were subjected to Calcein-AM/PI staining for live/dead cell analysis. Although both GS and GHS systems exhibited minor cell death, GS had significantly higher dead cells than GHS in the corresponding period. In GHS, only a few dot-like dead cells (marked by yellow triangles) were observed in the core region of the spheroids, and cell death appeared to initiate from the core and gradually extend toward the periphery of the spheroids (Figure 4B). Furthermore, compared to GHS, cells in GS appeared more loosely packed, and the edges were more blurred, which may indicate decreased cell viability. These findings further confirm that the co-culture of HUVECs promotes cell viability within the GHS.

### 3.4 HUVECs enhance the maintenance of cell viability within the GHS system

**FIGURE 5:**
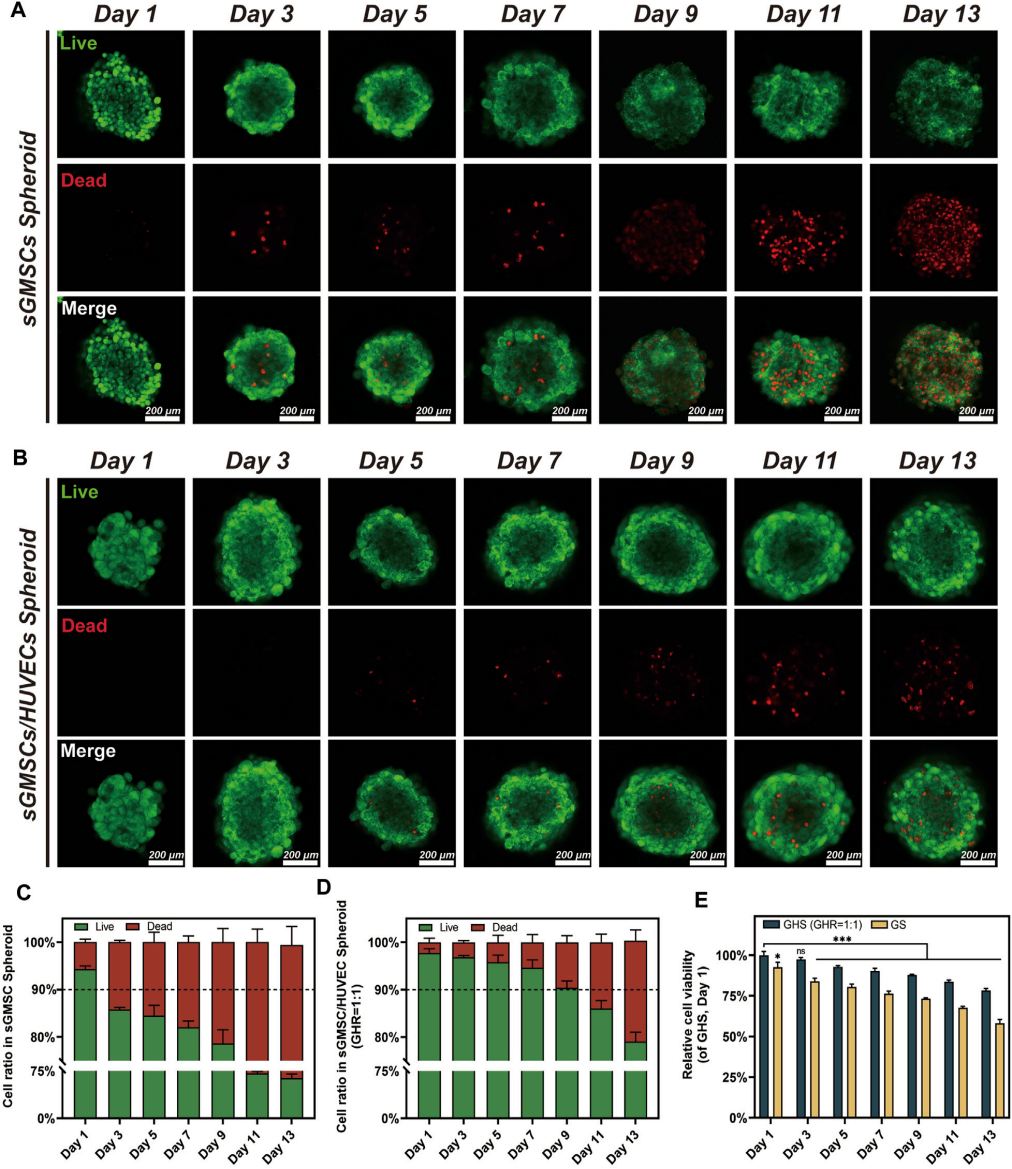
Maintenance of cell viability within GHS and GS systems. **(A, B)**. Scale bar, 200 μm) Observation of changes in cell viability during 2 weeks of osteogenic induction in GS and GHS. **(C, D)** Trends in live/dead cell ratios within GS and GHS during 2 weeks of osteogenic induction. **(E)** Relative cell viability of GS and GHS during 2 weeks of osteogenic induction were calculated by CCK-3D method. (Student’s two-tailed unpaired *t-test* and ANOVA, *p < 0.05, **p < 0.01, ***p < 0.001.)

As the induction period extended, dead cells became apparent within the GS and GHS, exhibiting a discernible trend of augmentation over time (Figures 5A, B). Furthermore, alterations in the morphology of GS spheroids, characterized by blurred boundaries and intercellular spaces (Figure 5A), were observed over time. By the third day of induction, the fraction of living cells within the GS was less than 90% (Figure 5C), with sporadic dead cells emerging in the core region of the spheroids (Figure 5A). By the 13th day, approximately 35.87% ± 3.93% of cells within the GS system were dead, with live cells accounting for approximately 63.54% ± 6.53%. Conversely, within the GHS, sporadic dead cells in the core region emerged between day 5 and 7 (Figure 5B). Despite the gradual increase in the count of dead cells over time, the live cell proportion in the GHS system did not fall below 90% until the 9–11th day of osteogenic induction (Figure 5D). On the 13th day, the GHS maintained a well-defined spherical morphology with tight and distinct intercellular characteristics; approximately 79.05% ± 1.97% of the cells were alive. Concurrently, the dead cell count was approximately 21.28% ± 2.27%, significantly lower than in the GS system at the equivalent time point (*t* = 5.593, *p = 0.005). The results from the CCK-3D assay demonstrated a higher relative cell viability within GHS at the corresponding time point compared to GS, with a statistically significant difference (Figure 5E). This trend aligns with alterations in the relative fluorescence intensity observed using Calcein-AM/PI staining.

These findings further corroborate the cell viability enhancement within the GHS system resulting from co-culturing with HUVECs and propose that the intercellular interactions between sGMSCs and HUVECs within the GHS system may underpin the long-term stability of the spheroids.

### 3.5 HUVECs enhance the osteogenic differentiation capacity of the GHS system

**FIGURE 6:**
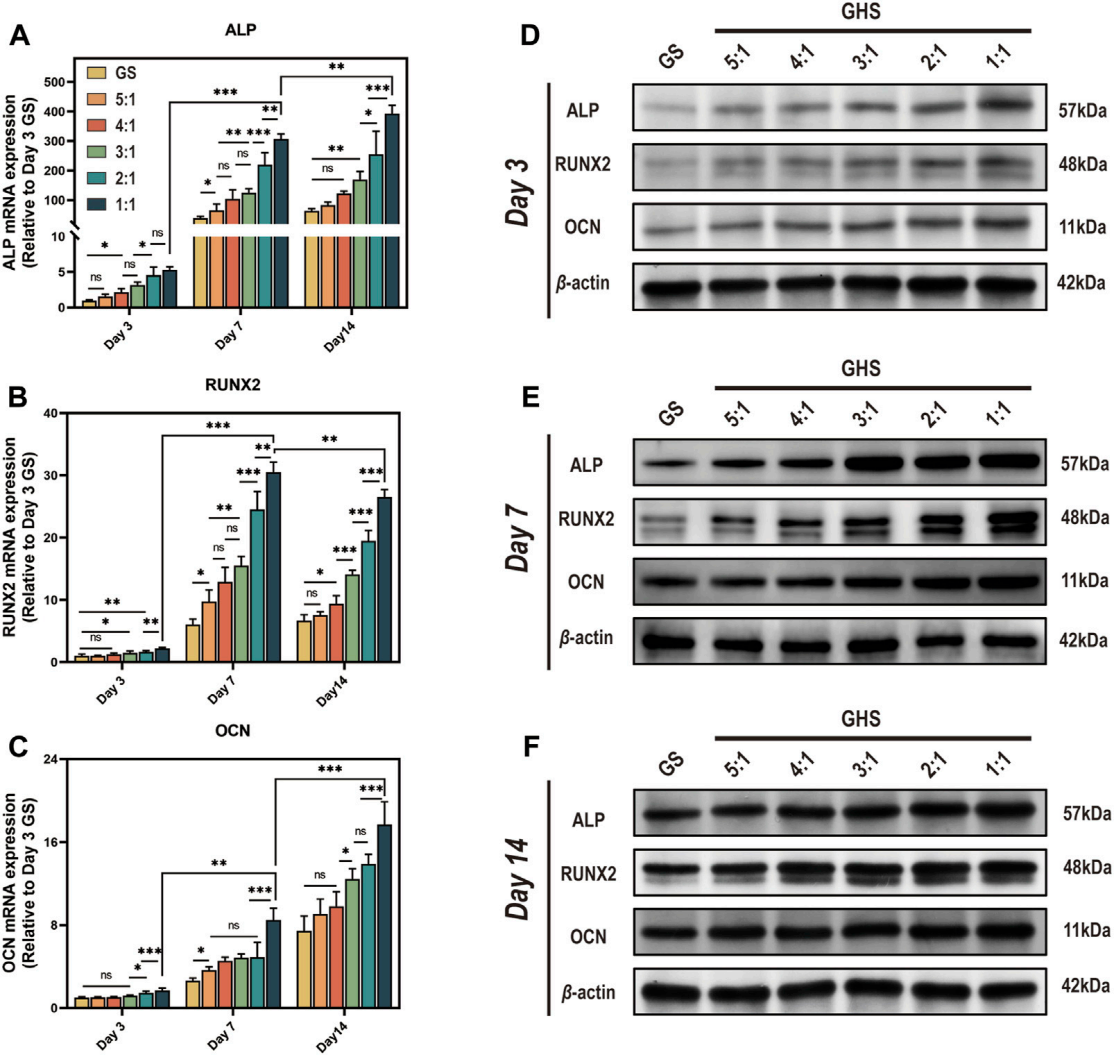
Enhancement of osteogenic differentiation capacity in GHS system by HUVECs. Results on day 3, 7, and 14 of osteogenic induction show: **(A-C)** qRT-PCR results indicate that co-culturing with HUVECs leads to increased relative mRNA expression of osteogenic differentiation genes ALP, RUNX2, and OCN in GHS at each time point. **(D-F)** Western blot analysis further confirms the relative upregulation of ALP, RUNX2, and OCN at the protein level in GHS. (ANOVA, “ns” represents non-significant, *p < 0.05, **p < 0.01, ***p < 0.001.)

After 3, 7, and 14 days of osteogenic induction, the mRNA and protein expression levels of each cell proportion group within the GHS system were significantly upregulated compared with the GS group, demonstrating statistical significance. Regarding the induction duration, as the osteogenic induction period prolonged, the mRNA and protein expression levels of the early osteogenic differentiation marker ALP and late osteogenic differentiation marker OCN exhibited significant increases. Specifically, on day 14, the mRNA expression levels of ALP and OCN were higher relative to days 3 and 7 (Figures 6A, C), with the protein expression levels showing a consistent trend with mRNA (Figures 6D–F). While the relative mRNA expression of the early osteogenic differentiation marker RUNX2 peaked on day 7 (Figure 6B). From the perspective of cell ratio within the GHS, on day 3 of osteogenic induction, the difference in osteogenic differentiation marker expression between the GS and GHS groups of each cell ratio was not prominent, particularly when the proportion of HUVECs cells was low (e.g., GHR of 5:1, 4:1, and 3:1). However, with higher HUVECs proportions, expression levels of the osteogenic marker were significantly higher than those of the GS group. Moreover, as the proportion of HUVECs within the GHS system increased, the expression levels of ALP, RUNX2, and OCN correspondingly increased with prolonged induction time. These results imply that co-culturing HUVECs with sGMSCs could enhance the osteogenic differentiation capacity of sGMSCs within the GHS system.

### 3.6 HUVECs compensate for the deficiencies in angiogenic differentiation of the GS system

**FIGURE 7:**
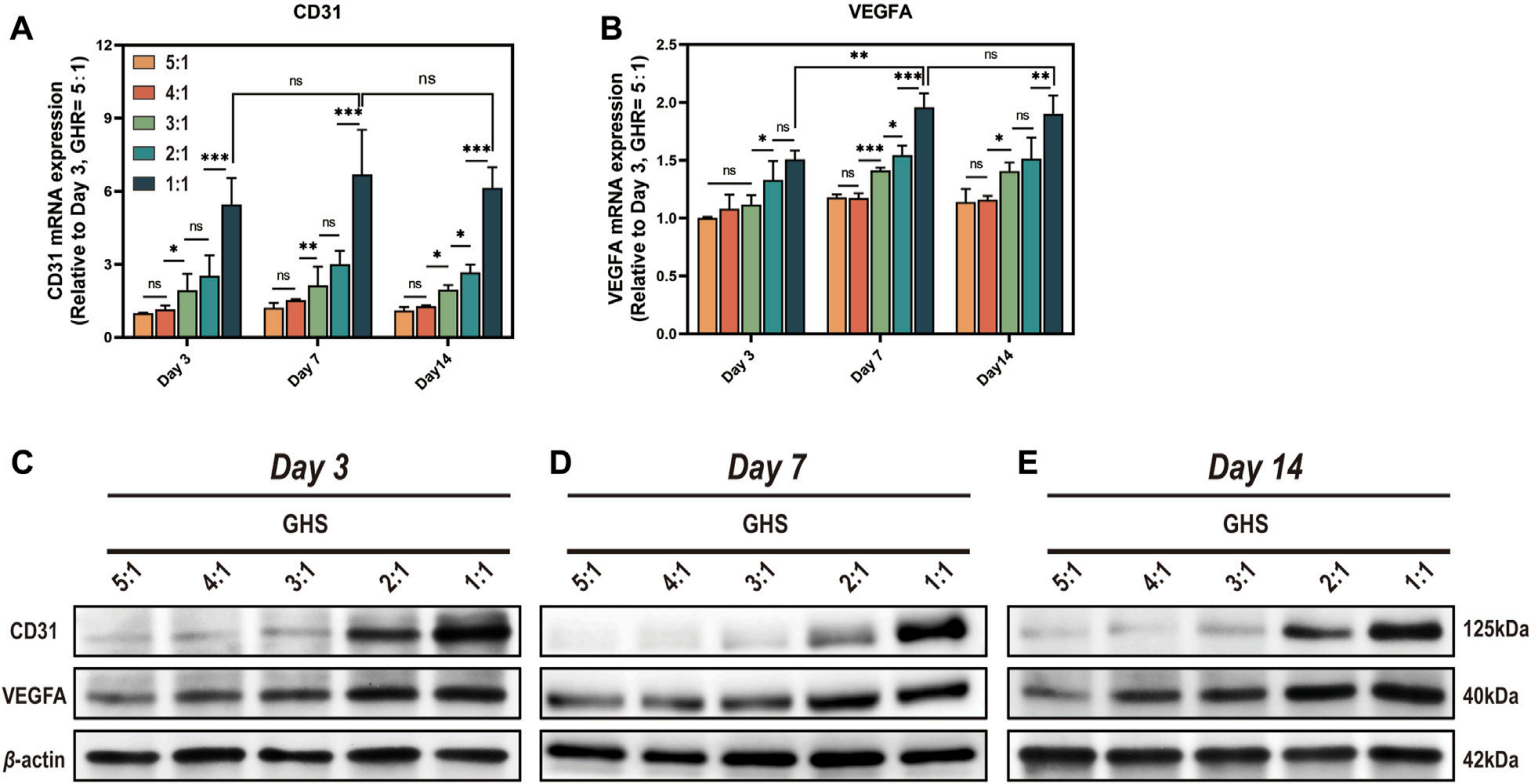
Complementation of angiogenic differentiation deficiency in GS by co-cultured with HUVECs. On day 3, 7, and 14 of osteogenic induction, GHS with different GHR exhibit distinct expression of angiogenic differentiation markers **(A, B)**. qRT-PCR results demonstrate that introducing HUVECs in constructing GHS initiates the expression of angiogenic differentiation genes CD31 and VEGFA. **(C-E)** Western blot analysis shows that the protein expression trends of CD31 and VEGFA align with the mRNA results. (ANOVA, “ns” represents non-significant, *p < 0.05, **p < 0.01, ***p < 0.001.)

Since sGMSCs do not express angiogenic markers CD31 and VEGFA, we excluded the GS group for statistical analysis. From the perspective of cell ratio within the GHS, the relative expression of mRNA (Figures 7A, B) and protein (Figures 7C–E) of CD31 and VEGFA promoted as the proportion of HUVECs increased at each time point. When the GHR was 1:1, both mRNA and protein expressions of CD31 and VEGFA reached peaks, indicating a positive correlation between the expression of angiogenic markers by GHS and the proportion of HUVECs introduced into 3D co-culture. Regarding the induction duration, when the GHR was 1:1, there was no statistical significance in the mRNA expression levels of CD31 in each cell ratio group on days 3, 7, and 14 of osteogenic induction. The mRNA expression level of VEGFA on day 7 was significantly higher than that on day 3, but there was no statistical significance compared with day 14. Notably, the overall trend of protein expression levels of CD31 and VEGFA showed consistency across each time point (Figures 7C–E), indicating a weak time correlation of GHS angiogenic marker expression. However, as the cell ratio of co-cultured HUVECs increases, the angiogenic capacity of GHS increases and remains stable over time, contributing to the early vascularization and long-term cell viability maintenance of GHS.

## 4 Discussion

Traditional 2D osteo-induction methods lack sufficient interactions between cells and between cells and the ECM(Liu et al., 2022a), weakening the capability of MSCs to express specific differentiation markers (Yuste et al., 2021; Liu et al., 2022a). 3D culture of MSCs allows them to form cell spheroids through self-assembly, better simulating cell-cell and cell-ECM interactions and forming more complex cellular morphologies and structures to mimic the *in vivo* environment (Ryu et al., 2019). Compared to the 2D monolayer culture method, MSC spheroids cultivated in 3D demonstrate superior potential regarding cell vitality, multidirectional differentiation, and transplantability (Duval et al., 2017; Wolff et al., 2023). This increased potential is a key reason behind this study’s adoption of a 3D MSC spheroids system for bone regeneration.

The successful regeneration of bone tissue is largely contingent upon the appropriate selection of seed cells. MSCs derived from different sources within dental tissues exhibit varying biological characteristics and differentiation potential (Costela-Ruiz et al., 2022). GMSCs demonstrate a superior differentiation capacity to other dental tissue-derived stem cells, such as PDLSCs and DPSCs(Gao et al., 2014; Angelopoulos et al., 2018). They also display exceptional immunomodulatory and stemness-preserving properties (Dave et al., 2022). STRO1 is a stably expressed progenitor marker, and STRO1-positive stem cells often exhibit robust osteogenic and chondrogenic differentiation abilities and stemness maintenance (Chmilewsky et al., 2019; Ranga Rao and Subbarayan, 2019). In this study, we isolated sGMSCs with high expression of STRO1 from pGMSCs using the MACS technique to serve as seed cells for stable and efficient differentiation. Moreover, the vascular networks play a crucial role in bone tissue engineering, being essential for the delivery of nutrients and cytokines and for the transport of metabolic waste during the repair of bone defects (Zhou et al., 2021). The vascularization of grafts is integral to maintaining seed cell viability and encouraging the activation of regenerative differentiation potential (Schott et al., 2022). However, sGMSCs express minimal hematopoietic lineage markers, thus impeding the promotion of vascular network formation via induced differentiation *in vitro*. In contrast, HUVECs, recognized as mature endothelial cells, can produce the necessary factors and proteins for angiogenesis, subsequently fostering neointima formation (Khayat et al., 2017). During co-culture, the microvessels formed by HUVECs facilitate the supply of nutrients and oxygen to MSCs, thereby bolstering their growth and differentiation (Liu et al., 2022b; Li et al., 2022). Consequently, we hypothesize that incorporating HUVECs into the co-culture could compensate for the lower innate angiogenesis potential of the GS and potentially foster further differentiation of the stem cell components within the system.

To address the challenges of vascularization within sGMSC-based spheroid systems, this study incorporated HUVECs in varying GHR for 3D co-cultivation, thereby creating GHS with various cell proportions. The viability of cells within these spheroids was scrutinized, and the expression levels of osteogenic and angiogenic differentiation-associated factors were assessed. The results demonstrated that the integration of HUVECs in co-cultured spheroids prolonged cell viability and stability while triggering the expression of angiogenic differentiation factors CD31 and VEGFA. Interestingly, the expression levels of osteogenic differentiation markers such as OCN, RUNX2, and ALP were enhanced concurrently, suggesting a synergistic effect of HUVECs incorporation on osteogenic and angiogenic differentiation within GHS. Therefore, the co-cultured stem cell spheroid GHS developed based on this novel strategy can simultaneously promote angiogenesis and bone tissue regeneration, showing promise as potent graft candidates for augmenting bone tissue regeneration and vascularization in bone defect areas.

Although the GHS obtained *in vitro* in this study demonstrated robust cell activity and osteogenic differentiation capacity within 2 weeks, it is critical to note that bone regeneration is a protracted process in the clinic. Prolonged induction can adversely affect stem cell viability and differentiation capacity (Otte et al., 2013; Gu et al., 2016). In this study, we observed apoptosis of cells within the core region of both GS and GHS spheroids as induction time increased. This phenomenon can be attributed to hypoxia, nutrient deficiency, and the accumulation of metabolic waste (Liu et al., 2017; Murphy et al., 2017). With prolonged induction, GS underwent morphological changes characterized by looser cell arrangement and reduced cell-cell contacts, which may affect osteogenic and angiogenic differentiation. However, introducing HUVECs deferred apoptosis and morphological alterations within the GHS system. Furthermore, we found that even on the seventh day of induction, both GS and GHS retained relatively high cell viability, with over 80% of cells remaining viable. Therefore, we selected the seventh day of osteogenic induction for comparative analysis between GS and GHS with different GHR.

Comprehending the mechanism of action between HUVECs and sGMSCs within the GHS is critical for the translational application of GHS therapy. Our study noted that as the proportion of HUVECs within the GHS increases, there’s a correlated increase in the relative expression of osteogenic and angiogenic factors. This suggests that a higher proportion of HUVECs in GHS is more beneficial for osteogenic and angiogenic differentiation. Specifically, when the GHR was 1:1, the relative expression levels of osteogenic and angiogenic factors in GHS were the highest, confirming this observation. We speculate that the following changes in cellular behaviors may be attributed to the co-culturing effect.

(i) Alteration of the stem cell microenvironment or niche: Introducing the HUVECs’ ECM might modify the stem cell microenvironment or niche of MSCs, potentially influencing cell morphology, function, and signaling (Wagers, 2012; Kobolak et al., 2016; Liu et al., 2022a). Similarly, the ECM of sGMSCs might also transform the cellular microenvironment, thereby enhancing the survival of HUVECs.
(ii) Cytokine interaction: Throughout the differentiation of MSCs into osteoblasts, cytokines like BMP2, bFGF, and PDGF are secreted. These cytokines foster the growth, migration, and angiogenesis of HUVECs(Liu et al., 2019; Zhou et al., 2021). Conversely, HUVECs secrete VEGFA, bFGF, TGF-β, HIF-1α, and IGF-1, collectively promoting the proliferation and osteogenic differentiation of MSCs(Chiesa et al., 2020; Yao et al., 2020; You et al., 2023). This reciprocal interaction between the 2 cell types synergistically boosts cell viability within the GHS system and stimulates the release of osteogenic and angiogenic factors.
(iii) Cellular crosstalk: Murphy et al. have demonstrated the potent paracrine regulatory function of MSCs(Murphy et al., 2013). The interaction between sGMSCs and HUVECs might occur either through direct cell-to-cell contact or indirect interaction via the transfer of extracellular vesicles (EVs). This process can stimulate multiple signaling pathways, thereby enhancing osteogenic differentiation and initiating the release of angiogenic factors within the GHS. Hsu et al. demonstrated through *in vivo* experiments that 3D MSCs/ECs spheroids could promote angiogenesis by recruiting host endothelial cells and pericytes through paracrine signaling and directly forming new blood vessels (Hsu et al., 2021).
(iv) Antioxidant Properties of HUVECs: Reactive oxygen species (ROS) are highly reactive oxygen molecules generated due to a redox state imbalance and can detrimentally impact cell growth and metabolism, thereby influencing MSCs differentiation and function (Denu and Hematti, 2016). Osteogenic induction demands a certain duration, during which MSCs under induction conditions can accumulate ROS due to prolonged high metabolic levels, leading to impaired osteogenic differentiation ability (Yang et al., 2018). HUVECs can enhance nutrient and oxygen supply to MSCs during culture and produce antioxidant factors such as Superoxide Dismutase (SOD) and Glutathione Peroxidases (GPx), which can mitigate ROS accumulation through degradation (La Sala et al., 2016). This mechanism might explain why co-cultured GHS can sustain cell viability and differentiation capacity for extended periods.
(v) Mitochondrial Transfer in MSCs: Mitochondrial function is essential in cellular homeostasis and metabolism control. During osteogenic induction, MSCs may engage in intercellular mitochondrial transfer via Gap junction channels (GJCs), cell fusion, EVs, tunneling nanotubes (TNTs), and other mechanisms, consequently promoting osteogenic differentiation and enhancing cell viability in MSCs(Hsu et al., 2016; Li et al., 2017; Zheng et al., 2020; Yan et al., 2021; Wei et al., 2023). Intriguingly, our results corroborate that introducing HUVECs bolsters cellular ATP activity within GHS and significantly augments osteogenic differentiation. Given the intimate connection between ATP production and mitochondrial activity, the amplified cell viability and osteogenic differentiation capacity observed in GHS might be associated with mitochondrial transfer in MSCs.

The aforementioned cellular behaviors do not operate in isolation but in mutually intersecting and overlapping interactions. In essence, within the osteogenic induction culture milieu, the co-culture of these cell types triggers various signaling pathways beneficial for maintaining cellular homeostasis within the GHS system and fostering the osteogenic and angiogenic differentiation of sGMSCs. Although our results have corroborated this point, further research is required to delineate the underlying mechanisms.

Stem cell therapy in bone tissue engineering is often integrated with traditional surgical techniques such as bone grafting and guided tissue regeneration (GTR) (Chen et al., 2016). The development of GHS presents a potential graft candidate for these methodologies. This study investigated the cell ratio and culture duration of 3D GHS for bone tissue engineering *in vitro*, thereby unveiling its therapeutic potential in vascularizing bone defects. However, certain challenges must be overcome before progressing toward clinical application. First, the transplanted cell spheroids need to thrive in a suitable microenvironment. Factors such as hypoxia, nutrient deficiency, and immune response often result in low survival rates and functionality of the transplanted cells (Ho et al., 2016; Gionet-Gonzales et al., 2021). Secondly, the transplanted cell spheroids need precise positioning and fixation at the treatment site to exert their therapeutic effects. However, the migration and distribution of cells *in vivo* are subject to various influences, and in the absence of scaffold materials, cell leakage and consequential loss are more likely to occur (Griffin et al., 2022). Therefore, future research should address these challenges to enhance the survival rate and therapeutic efficacy of transplanted cell spheroids.

Given the time and budget constraints, our study focused on constructing and investigating the osteogenic and angiogenic differentiation potential of GHS *in vitro*. Moving forward, our primary objective is to standardize the 3D GHS construction protocol for bone tissue engineering, with particular emphasis on developing biomimetic scaffold materials to encapsulate and stabilize GHS, aiming to uphold their long-term survival and *in vivo* differentiation functionality. The feasibility of GHS transplantation will be corroborated through animal models of periodontal bone defects, with subsequent optimization of the therapeutic potential of 3D GHS. Besides, further research is warranted to clarify the specific mechanisms through which HUVECs enhance the osteogenic and angiogenic differentiation of 3D GHS.

## 5 Conclusion

In this study, we successfully addressed the deficiency in angiogenic differentiation of spheroids composed of sGMSCs by co-culturing them with HUVECs, resulting in the formation of GHS. Furthermore, we discovered that HUVECs enhanced the osteogenic differentiation potential of sGMSCs within the co-culture spheroid system. GHS demonstrated superior *in vitro* osteogenic and angiogenic differentiation capacities to those comprised solely of sGMSCs. Additionally, the introduction of HUVECs in the co-culture system improved and prolonged the maintenance of cell viability within the cell spheroids. Our results suggest that 3D co-culture with HUVECs provides a feasible strategy for enhancing osteogenic and angiogenic differentiation within GS, offering a promising avenue for MSC spheroid-based bone tissue engineering.

## Data Availability

The original contributions presented in the study are included in the article/Supplementary material, further inquiries can be directed to the corresponding author.
